# Incidence of prostate and urological cancers in England by ethnic group, 2001-2007: a descriptive study

**DOI:** 10.1186/s12885-015-1771-2

**Published:** 2015-10-21

**Authors:** Mahiben Maruthappu, Isobel Barnes, Shameq Sayeed, Raghib Ali

**Affiliations:** 1 Green Templeton College, University of Oxford, Oxford, OX2 6HG UK; 2 Cancer Epidemiology Unit, University of Oxford, Richard Doll Building, Roosevelt Drive, Oxford, OX3 7LF UK; 3 New York University Abu Dhabi, Abu Dhabi, PO Box 129188, United Arab Emirates

**Keywords:** Urological, Cancer, Ethnic, Prostate, Incidence, Kidney, Bladder, Testicular

## Abstract

**Background:**

The aetiology of urological cancers is poorly understood and variations in incidence by ethnic group may provide insights into the relative importance of genetic and environmental risk factors. Our objective was to compare the incidence of four urological cancers (kidney, bladder, prostate and testicular) among six **‘**non-White**’** ethnic groups in England (Indian, Pakistani, Bangladeshi, Black African, Black Caribbean and Chinese) to each other and to Whites.

**Methods:**

We obtained Information on ethnicity for all urological cancer registrations from 2001 to 2007 (n = 329,524) by linkage to the Hospital Episodes Statistics database. We calculated incidence rate ratios adjusted for age, sex and income, comparing the six ethnic groups (and combined **‘**South Asian**’** and **‘**Black**’** groups) to Whites and to each other.

**Results:**

There were significant differences in the incidence of all four cancers between the ethnic groups (all p < 0.001). In general, ‘non-White’ groups had a lower incidence of urological cancers compared to Whites, except prostate cancer, which displayed a higher incidence in Blacks. (IRR 2.55) There was strong evidence of differences in risk between Indians, Pakistanis and Bangladeshis for kidney, bladder and prostate cancer (p < 0.001), and between Black Africans and Black Caribbeans for all four cancers (p < 0.001).

**Conclusions:**

The risk of urological cancers in England varies greatly by ethnicity, including within groups that have traditionally been analysed together (South Asians and Blacks). In general, these differences are not readily explained by known risk factors, although the very high incidence of prostate cancer in both black Africans and Caribbeans suggests increased genetic susceptibility. g.

**Electronic supplementary material:**

The online version of this article (doi:10.1186/s12885-015-1771-2) contains supplementary material, which is available to authorized users.

## Background

Urological cancers account for about 14 % of cancers diagnosed globally and more than a fifth of all cancers in Europe [1]. There is also significant international variation in incidence and the aetiology of urological cancers remains poorly understood. Identifying the extent of ethnic variation can contribute to our understanding of aetiology and assist in planning care for different ethnic groups. Unfortunately international comparisons are of limited value as registration systems vary in their quality; there are systematic variations between health systems and systematic biases exist in the way different populations access care [2].

The UK is a multi-ethnic society, with ‘non-White’ ethnic groups making up around 14 % of England's population in 2011. British (South) Asians - Indians, Pakistanis and Bangladeshis—form the largest group of about 6 %, and British Blacks - Black Africans (mainly from Nigeria, South Africa, Ghana and Somalia) and Black Caribbeans (predominantly from Jamaica)—are second at about 3 %, with Chinese (mainly from Hong Kong) about 1 % [3]. Studies have shown South Asians in the U.S. to have lower rates of kidney, bladder, prostate and testicular cancer than Whites and Blacks [4]. South Asians, however, are a heterogeneous group with varied socio-cultural practices and the risk of urological cancers within each individual ethnic group is unknown. Further, these data do not consider socioeconomic status, and are therefore subject to under-reporting in ethnic groups with decreased access to care [5].

Although it has long been known that there are differences in the incidence of many cancers by ethnic group [6] and in access to healthcare (including screening) due to socioeconomic disadvantages [7], studies of cancer incidence in ethnic groups in the UK have been of limited accuracy in the past due to the incomplete ethnicity data held by cancer registries. Various techniques have been used to try and overcome this problem, including using country of birth, the calculation of proportional incidence ratios and assigning ethnicity on the basis of name [8–10]. However, all these methods have significant limitations and the most accurate method is to use self–assigned ethnicity (as has been done in the census since 1991) which allows us to use the same method of assigning ethnicity in the numerator and denominator.

From 1995, self-assigned ethnicity has been recorded in the National Health System’s Hospital Episodes Statistics (HES) database, and HES records can now be linked to cancer registrations, providing more reliable information on ethnicity [11]. Although the recording of routine ethnicity data in primary care is still limited [12], hospital data is much better and has improved markedly in the last 20 years, with the percentage of missing ethnicity values falling from 35 % in 1998 to less than 10 % by 2009 [13]. In England, consistency of diagnostic methods, reporting and registration procedures across the entire health system removes significant biases intrinsic to databases in many other countries.

Our objective was to compare the incidence of kidney, bladder, prostate and testicular cancer amongst ethnic groups (Indian, Pakistani, Bangladeshi, Black African, Black Caribbean and Chinese) in England, to each other and to Whites.

## Methods

The methods used in this study were broadly the same as those described in our previous studies [14–16] and are summarized below.

### Data collection

Data were obtained from the National Cancer Intelligence Network (NCIN) for all cancer registrations from January 2001 to December 2007 in England: cancer site coded to the International Classifications of Diseases, 10th Revision (ICD-10) [17]; morphology coded to the International Classifications of Diseases of Oncology, 2nd and 3rd Revisions (ICD-O-2 and ICD-O-3) [18, 19]; deprivation assessed from the income domain of the Index of Multiple Deprivation 2007 (IMD 2007) [20]; age at diagnosis of cancer; sex and ethnicity. To determine population incidence data, mid-year population estimates produced by the Office of National Statistics (ONS) from 2001 to 2007 were used, stratified by age, sex and ethnicity. Population data stratified by national quintiles of the income domain were provided by ONS based on the 2001 census and the same distributions applied to population data by age, sex and ethnicity for the 2001-2007 mid-year population estimates.

### Classification of ethnicity

NCIN obtained the self-assigned ethnicity for each cancer registration by record linkage to the Hospital Episodes Statistics (HES) database. If a cancer registration could not be linked to HES, or if ethnicity data were missing on the HES database, then ethnicity was assigned using information recorded in the cancer registry data. Prior to April 2001, ethnicity was coded both by HES and by cancer registries using the classification system of the 1991 Census. After April 2001, the codes were amended to those of the 2001 Census, although 1991 ethnicity codes were accepted until 2003. For these analyses, we classified ethnicity as White (White from the 1991 Census and White British from the 2001 Census), Indian, Pakistani and Bangladeshi, (with the three groups combined to form the category ‘South Asian’), Black African, Black Caribbean (again both combined to form the category, ‘Black’) and Chinese. (Sri Lankans are not recorded as a separate ethnic group in the census or HES data and so are not included in our analysis.)

### Classification of cancers

Cancers were classified as cancers of the prostate (ICD-10 code C61), testes (C62), kidney (C64, C65, C66 and C68) and bladder (C67).

### Statistical analyses

We estimated age standardised rates (ASRs) of each cancer per 100,000 person-years for all ethnic groups using direct standardisation to the 1960 Segi world population [21], with age at diagnosis of cancer being classified into 6 categories: <40, 40–49, 50–59, 60–69, 70–79, and ≥ 80 years. We used Poisson regression to estimate incidence rate ratios (IRRs) comparing each ethnic group, and the two combined categories of South Asian and Black, to Whites adjusting for age, sex (where appropriate) and deprivation.

When comparing South Asians and Blacks to Whites, we present results as RRs and 99 % confidence intervals (CIs). When comparing the individual ethnic groups, results are presented as IRRs and 99 % floating confidence intervals (FCIs). FCIs were calculated using the method of floating absolute risks [22] and enable valid comparisons between any two ethnic groups, even if neither one is the baseline. We calculated 99 % CIs because of multiple tests performed across ethnic groups.

We performed pre-specified subgroup analyses by sex, where appropriate, and by tumor type, which were grouped according to morphology. Specifically cancers of the prostate were grouped as adenocarcinoma (ICD-O-3 codes 8140, 8141 8143, 8147, 8211, 8251, 8255, 8260–82633, 8310, 8480, 8481, 8503, 8570–8574) and other tumors; cancer of the testes as seminoma (9060–9062, 9064) and non-seminomatous (9065–9102); cancer of the kidney as renal cell carcinoma (8050, 8140,8260, 8270, 8280–8312, 8316–8320, 8340–8344) and other; and cancer of the bladder as transitional (8050,8120–8122,8130–8131) and other. We also did a pre-specified subgroup analysis by age for prostate cancer, with cases divided into those aged under 50 and those aged 50 or above. We did not analyse the other cancers by age as case numbers were too low.

Tests of heterogeneity of IRRs between ethnicities, either overall or restricted to South Asians or Blacks, were performed using likelihood χ^2^ ratio tests. The test of heterogeneity of RRs between pre-specified subgroups was performed for South Asians, Blacks and Chinese using a χ^2^ contrast test.

### Sensitivity analysis

Because ethnicity information was not complete for all registered cancers, we used multiple imputations to assess the effect the missing values of ethnicity had on our results. We generated 40 datasets with imputed values of ethnicity using a multinomial logistic regression model where the predictor variables were age, deprivation (income) and site of cancer. We performed our primary analysis examining the effect of ethnicity on cancer for each dataset. The resulting IRRs were combined using Rubin’s combination rules [23].

We performed all analyses using Stata V.12 and R statistical software packages.

### Graphical presentation of results

Where results are presented in the form of plots, we represent IRRs for each ethnic group by squares and their corresponding 99 % FCIs by straight lines. For the combined ‘South Asian’ and ‘Black’ group, we show IRRs as open diamonds, whose horizontal extent indicates the 99 % CI. We placed a dashed vertical line at the value of the IRRs for all South Asians and for all Blacks.

### Comparison to rates in countries of origin

We also compared the ASRs for each ethnic group in England to rates from their country or region of origin using data from the GLOBOCAN database [1]. For Blacks, we used GLOBOCAN estimates for Sub-Saharan Africa and the Caribbean; there are no population based cancer registries in their main countries of origin.

This study was approved by the Oxford Research Ethics Committee.

## Results

Table 1 shows socio-demographic information from the 2001 census for Whites, Indians, Pakistanis, Bangladeshis, Black Africans, Black Caribbeans and Chinese. All six groups are, on average, younger than Whites and all except Chinese are also poorer, with Pakistanis, Bangladeshis and Black Africans being the most deprived.

**Table 1:** Comparison of demographic characteristics by ethnic group in England in 2001 using data from the 2001 census

Ethnic group		White		Indian		Pakistani		Bangladeshi		Black African		Black Carribean		Chinese	
		N	(%)	N	(%)	N	(%)	N	(%)	N	(%)	N	(%)	N	(%)
Census data for 2001															
Total population		42,747,136	100	1,028,546	100	706,539	100	275,394	100	475,938	100	561,246	100	220,681	100
Sex	Male	20,828,644	48.7	511,204	49.7	358,043	50.7	138,972	50.5	229,103	48.1	259,881	46.3	105,913	48.0
Age	<50	27,665,393	64.7	828,200	80.5	625,118	88.5	248,841	90.4	432,985	91.0	426,424	76.0	184,675	83.7
	50+	15,081,743	35.3	200,346	19.5	81,421	11.5	26,553	9.6	42,953	9.0	134,822	24.0	36,006	16.3
Deprivation	Low income	7,305,527	17.1	347,098	33.7	455,710	64.5	198,884	72.2	277,858	58.4	292,537	52.1	49,427	22.4
	Middle income	26,315,786	61.6	563,939	54.8	222,038	31.4	69,325	25.2	177,234	37.2	245,103	43.7	123,994	56.2
	High income	9,125,823	21.3	117,509	11.4	28,791	4.1	7,185	2.6	20,846	4.4	23,606	4.2	47,260	21.4
Country of Birth	United Kingdom	41,911,150	98.0	472,545	45.9	387,198	54.8	127,902	46.4	161,050	33.8	324,764	57.9	62,209	28.2
	Other	835,986	2.0	556,001	54.1	319,341	45.2	147,492	53.6	314,888	66.2	236,482	42.1	158,472	71.8

Table 2 shows the number of cancer registrations by ethnic group, and missing ethnicity values for each cancer. In total there were 329,524 urological cancer registrations and ethnicity information was missing in 81,767 (24.8 %) cases.

**Table 2:** Distribution of registered cancers from 2001-2007 in England by ethnic group and missing ethnicity values (percentages in brackets)

Cancer	White		Indian		Pakistani		Bangladeshi		Black African		Black Caribbean		Chinese		All other ethnic groups		No ethnicity recorded		Total
Prostate	132278	(62.5)	934	(0.4)	491	(0.2)	90	(0.0)	861	(0.4)	3185	(1.5)	226	(0.1)	10624	(5.0)	63068	(29.8)	211757
Testes	50133	(81.2)	223	(0.4)	117	(0.2)	42	(0.1)	69	(0.1)	186	(0.3)	62	(0.1)	3135	(5.1)	7762	(12.6)	61729
Kidney	7890	(65.7)	88	(0.7)	65	(0.5)	8	(0.1)	17	(0.1)	28	(0.2)	18	(0.2)	831	(6.9)	3064	(25.5)	12009
Bladder	32775	(74.4)	246	(0.6)	170	(0.4)	58	(0.1)	146	(0.3)	239	(0.5)	57	(0.1)	2465	(5.6)	7873	(17.9)	44029
All four cancers	223076	(67.7)	1491	(0.5)	843	(0.3)	198	(0.1)	1093	(0.3)	3638	(1.1)	363	(0.1)	17055	(5.2)	81767	(24.8)	329524

**Fig. 1:**
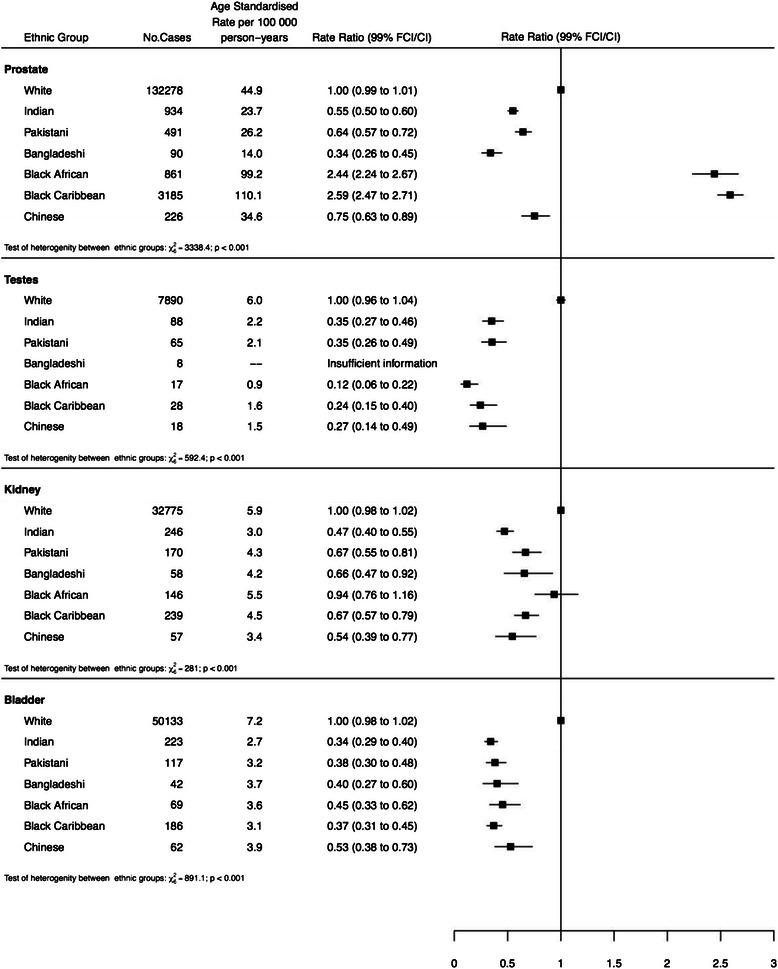
Age-standardised incidence rates and rate ratios (adjusted by age, sex and income) for the four urological cancers by individual ethnic group compared to Whites

Figure 1 shows the overall age-standardised incidence rates and rate ratios, adjusted by age, sex and income, for the four urological cancers by individual ethnic group compared to Whites. For all four cancers, there is significant heterogeneity between ethnic groups (p < 0.001), with a lower incidence for all ethnic groups compared to Whites, for all cancers except prostate cancer, where Blacks had by far the highest incidence.

**Fig. 2:**
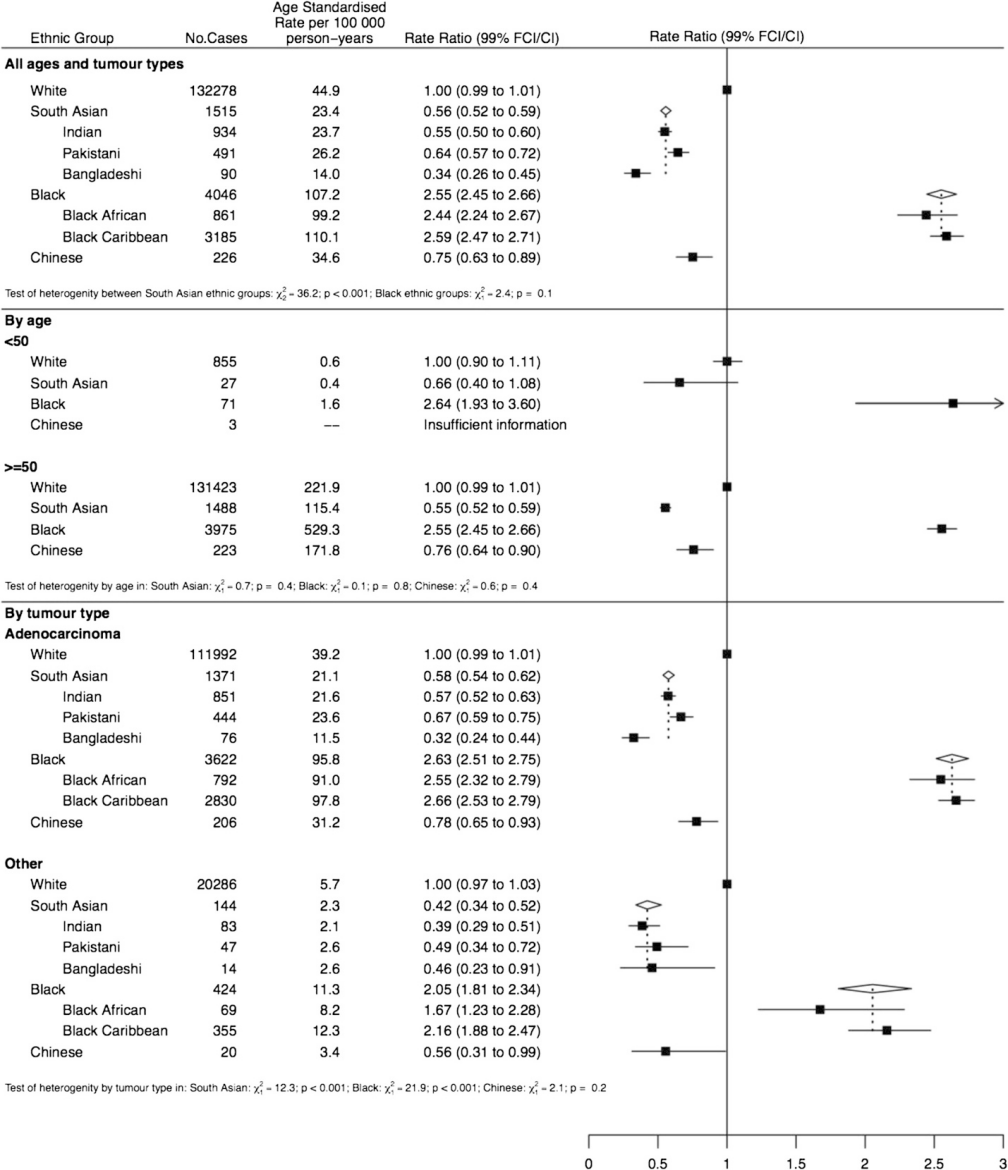
Age-standardised incidence rates and rate ratios (adjusted by age and income) for prostate cancer by ethnic group. Subgroups show rates and rate ratios subdivided by age and morphology (adenocarcinoma and other)

For kidney cancer (Fig. 2), the overall incidence in Chinese and South Asians was about half that in Whites, with risk in Indians significantly lower than in Pakistanis and Bangladeshis. (IRRs of 0.47, 0.67 and 0.66 respectively, P < 0.001). The incidence in Blacks was also lower than Whites with higher rates in Black Africans than Black Caribbeans. (IRRs of 0.94 and 0.67 respectively, P = 0.002). These trends were maintained in subgroup analyses by tumour type. Across all ethnicities, risk was higher in men than women but the relative risk compared to Whites was similar in men and women for all non-White groups.

**Fig. 3:**
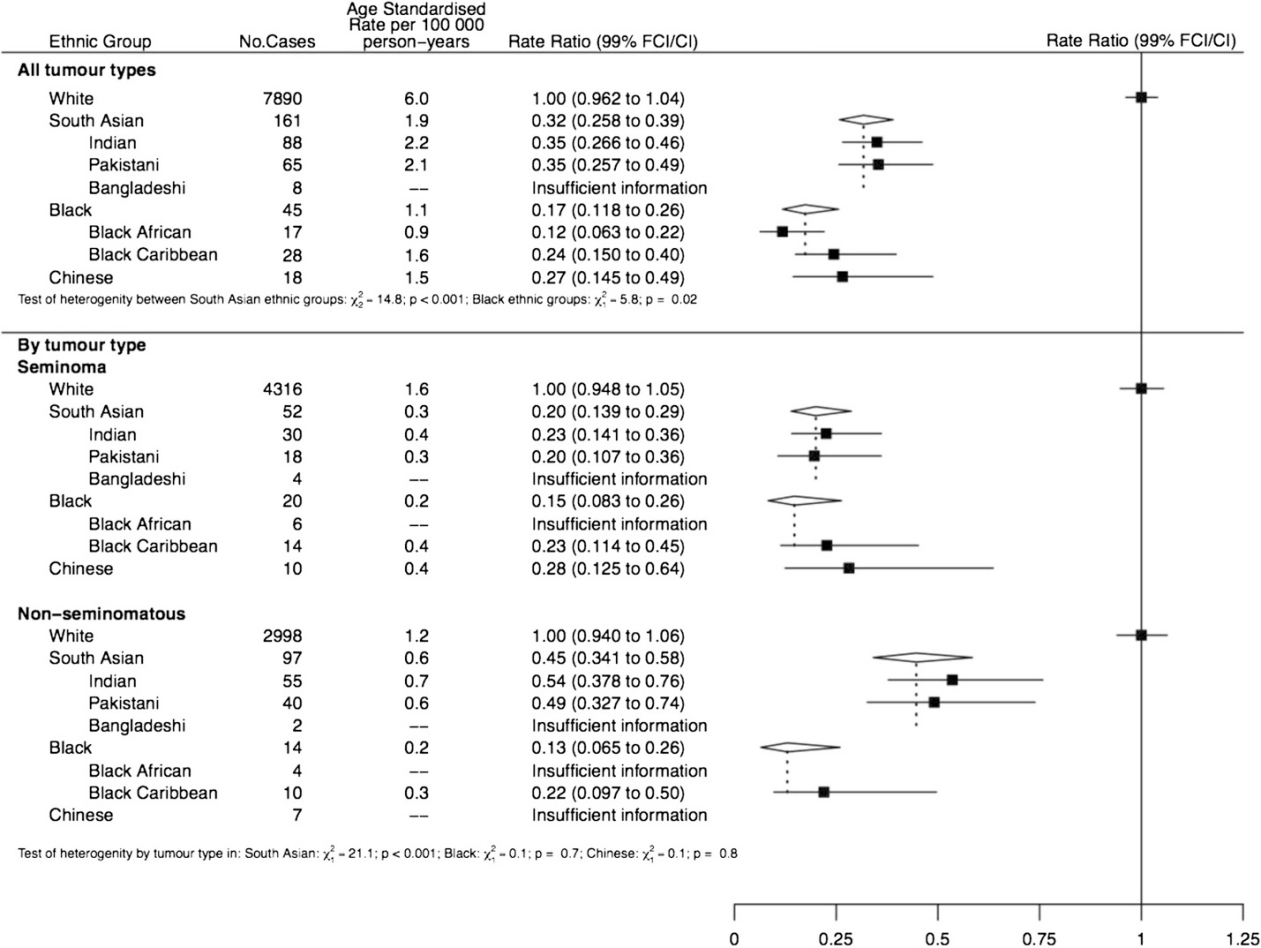
Age-standardised incidence rates and rate ratios (adjusted by age and income) for testicular cancer by ethnic group. Subgroups show rates and rate ratios subdivided by morphology (seminoma and non-seminomatous)

For bladder cancer (Fig. 3), the overall incidence in South Asians and Blacks was nearly two thirds lower than in Whites with no significant difference between Indians, Pakistanis and Bangladeshis, or between Black Africans and Black Caribbeans. The risk in Chinese was about half that of Whites. These trends were maintained in subgroup analyses by tumour type. Across all ethnicities, risk was higher in men than women but the relative risk compared to Whites was similar in men and women for all non-White groups.

**Fig. 4:**
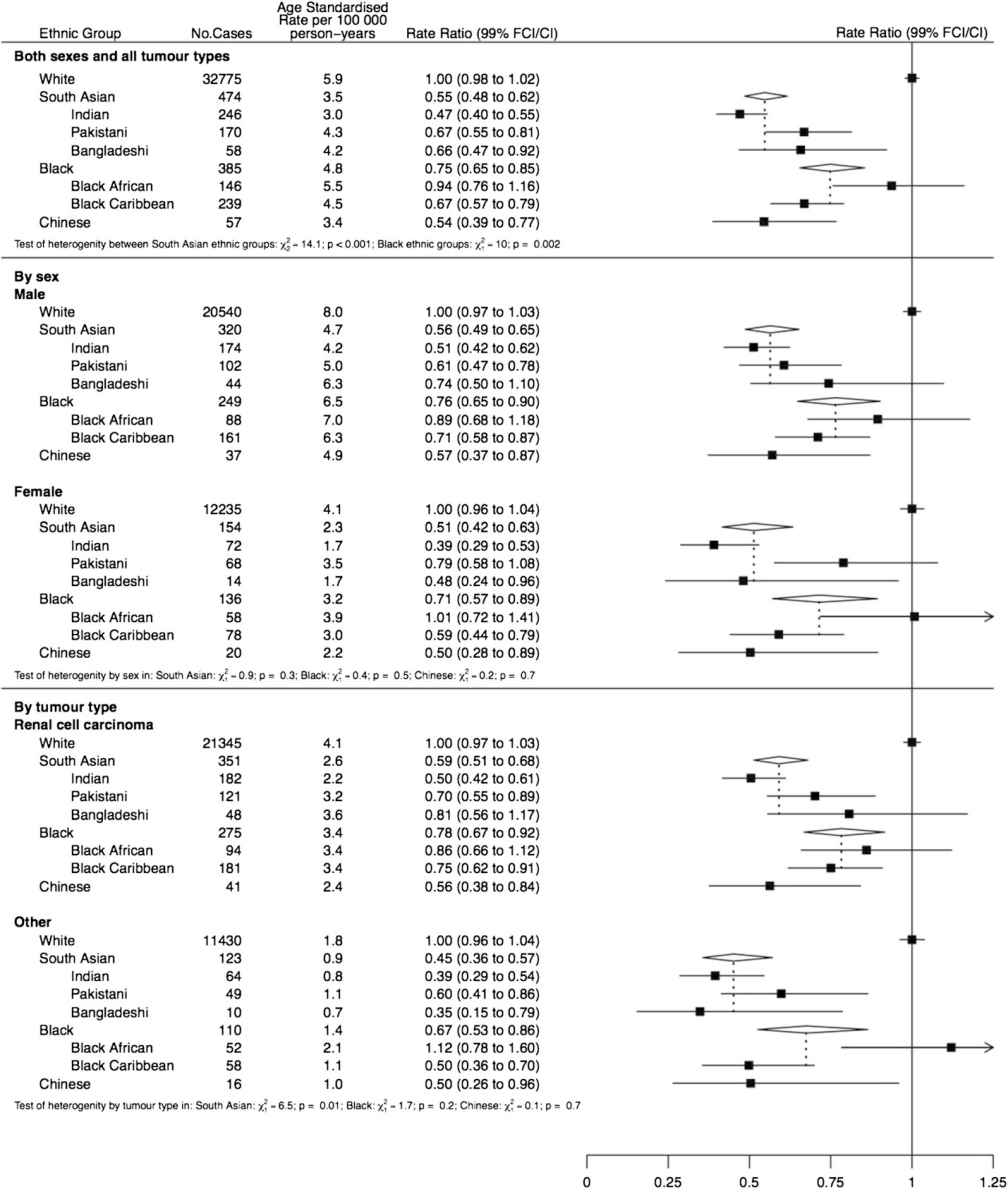
Age-standardised incidence rates and rate ratios (adjusted by age, sex and income) for kidney cancer by ethnic group. Subgroups show rates and rate ratios subdivided by sex and morphology (renal cell cancer & other)

For prostate cancer (Fig. 4), the overall incidence in South Asians was almost half that in Whites with substantial differences between Indians, Pakistanis and Bangladeshis (IRRs of 0.55, 0.64 and 0.33 respectively, P < 0.001) with Chinese also having a lower incidence than Whites. The incidence in both Black Caribbeans and Black Africans was more than double that of Whites. These trends were confirmed in subgroup analyses by both age and tumour type; Black Caribbeans and Black Africans displayed the highest incidence in both those aged less than and greater than 50 and in both adenocarcinoma and ‘other’ types of prostate cancer.

**Fig. 5:**
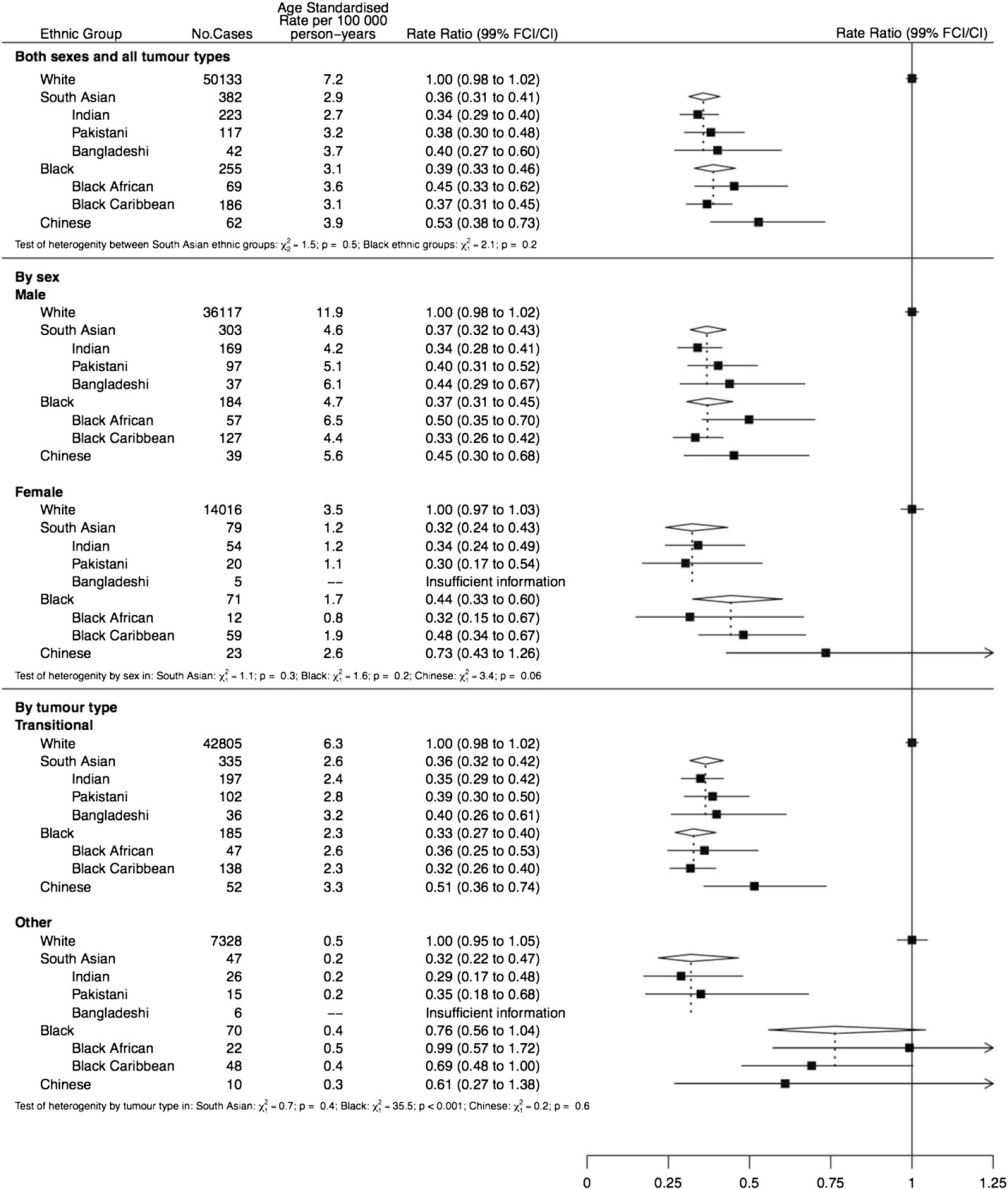
Age-standardised incidence rates and rate ratios (adjusted by age, sex and income) for bladder cancer by ethnic group. Subgroups show rates and rate ratios subdivided by sex and by morphology (transitional cell cancer & other)

For testicular cancer (Fig. 5), incidence in all ethnic groups was much lower than in Whites, about a third in South Asians and Chinese with blacks having the lowest incidence and lower rates in Black Africans than Black Caribbeans. These trends were maintained in subgroup analyses by tumour type and also showed that South Asians have a higher incidence of non-seminomatous tumours compared to seminomas.

### Sensitivity analysis

In the sensitivity analysis which assigned missing values using multiple imputations, results very similar to those shown in Fig. 1 were obtained, as shown in Additional file 1: figure S1 (online).

### Comparison to rates in countries of origins

Table 3 compares international data on age standardised incidence rates from GLOBOCAN. Across all ethnicities, ASRs for kidney, prostate and testicular cancers were lower in their countries of origins. Bladder cancer incidence was higher in respective countries of origins, than in comparative ethnicities in England. For all ethnicities, in both England and countries of origins, the incidence of kidney, bladder, prostate and testicular cancers was less than in Whites, except for prostate cancer in Caribbeans which was higher in the Carribean than in Whites.

**Table 3:** Age-standardised incidence rates for urological cancers by ethnic group in England compared to rates in country of origin using estimates from Globocan. (sub-Saharan Africa for Black Africans, Caribbean for Black Caribbeans)

		England		Incidence from Globocan
Cancer Site	Ethnicity	Cases	ASR	ASR
Prostate	White	132278	44.9	
	Indian	934	23.7	3.7
	Pakistani	491	26.2	5.2
	Bangladeshi	90	14.0	1.9
	Black African	861	99.2	21.2
	Black Caribbean	3185	110.1	71.1
	Chinese	226	34.6	4.3
Testes	White	50133	11.6	
	Indian	223	6.8	0.6
	Pakistani	117	9.5	0.9
	Bangladeshi	42	6.3	1.0
	Black African	69	8.9	0.4
	Black Caribbean	186	6.4	0.7
	Chinese	62	9.8	0.4
Kidney	White	7890	5.9	
	Indian	88	3.0	1.1
	Pakistani	65	4.3	1.3
	Bangladeshi	8	4.2	1.1
	Black African	17	5.5	1.1
	Black Caribbean	28	4.5	2.7
	Chinese	18	3.4	2.8
Bladder	White	32775	7.2	
	Indian	246	2.7	2.8
	Pakistani	170	3.2	5.4
	Bangladeshi	58	3.7	2.6
	Black African	146	3.6	3.7
	Black Caribbean	239	3.1	5.8
	Chinese	57	3.9	5.5

## Discussion

We compared incidence rates for urological cancers in the main ‘non-White’ ethnic groups in England – South Asian (Indian, Pakistani and Bangladeshi), Black (African and Caribbean) and Chinese to Whites and to each other. There was considerable variation by ethnic group, even when age and socioeconomic factors were taken into account. Overall, urological cancers were diagnosed less often in all the ‘non-White’ ethnic groups, except prostate cancer in Blacks, which demonstrated a higher incidence than in Whites. Amongst South Asians, we demonstrated that the incidence of urological cancers substantially differed between Pakistanis, Bangladeshis and Indians, supporting the notion that South Asians should not be viewed as a single ethnic group. Similarly, large differences existed between Black African and Caribbean populations, highlighting the need to differentiate between these two groups.

The different patterns of cancer risk between ethnic groups suggest that our findings are unlikely to be due to systematic reporting biases in any of the ethnic groups compared to Whites. Our previous work using the same dataset showed increased risks of some gastrointestinal and haematological cancers in ethnic minority groups further supporting the absence of an under-reporting bias [14, 15]. Using self-assigned ethnicity is also more reliable than other measures of ethnicity (e.g. name analysis) as it uses the same measure of ethnicity in the numerator and denominator. We also adjusted for socioeconomic status, a potential confounder in studies of health and ethnicity, particularly when comparing Pakistanis, Bangladeshis and Blacks due to their higher levels of deprivation [5].

Our findings are consistent with current literature although we are not aware of any previous studies which present incidence by individual ethnic group for kidney, bladder and testes cancers. (which grouped all ‘Blacks’ and all ‘South Asians’ together.)

For renal cancer, a previous study showed that South Asians had a lower incidence of renal cell carcinoma compared with Whites, consistent with our results [24]. Smoking is a known risk factor for renal cell carcinoma [25] and our results are consistent with smoking prevalence by ethnic group [26]. Some reports have demonstrated higher incidence of renal cell carcinoma in African Americans than Whites [27], contrary to our findings. This discrepancy may be attributable to the fact that these studies were done in the USA where black populations may have different countries of origin.

For bladder cancer, previous reports indicate a lower incidence amongst south Asians [28]. Again, smoking is a known risk factor for bladder cancer [29] and our results are consistent with smoking prevalence by ethnic group with the exception of Chinese [26] who have the lowest smoking prevalence but the highest bladder cancer incidence. This may be due to genetic factors or other ethnicity-specific risk factors (e.g. dietary soy) which is frequently used in Chinese cuisine, has been associated with increased bladder cancer risk [30].

For testicular cancer, previous studies have also revealed a lower incidence amongst Asians and Blacks [31] consistent with our data. This may be due to inter-ethnic variations in environmental factors acting prenatally or early in childhood [32]. Cryptorchidism, a known risk factor of testicular cancer, may also vary between ethnicities, with reports of reduced incidence amongst black babies [33] but data is not available for South Asians.

For prostate cancer, studies in the USA have shown increased incidence in men with African ancestry, even after migration to areas of lower prevalence [34, 35]. Our finding that Black African and Black Caribbean’s demonstrated a higher incidence of prostate cancer than Whites is also consistent with previous UK studies [28, 31].

The specific cause of increased prostate cancer risk amongst Blacks is not known. A recent review of known risk factors for prostate cancer found limited environmental explanation for the racial differences in incidence [36]. Nonetheless, there have been a number of dietary factors which have been independently implicated, including intake of animal fats and products [37]. Increased risk amongst Blacks has also been attributed to genetic factors including: variants of the genes of the enzymes involved in androgen biosynthesis and metabolism, such as SRD5A2, CYP17, and CYP3A4; the C-genotypes CD14; NAT2 and NER genetic variants; and polymorphisms at 17q21 and 8q24 [38–42]. Although the much higher rates seen in Black Africans and Caribbean in the UK compared to their regions of origin is consistent with a change of environment causing the increase, the very low rates seen in sub-saharan Africa almost certainly underestimate the true incidence due to under-diagnosis and under-reporting. We also note that rates are generally higher in black populations everywhere—sub-Saharan Africa and the Caribbean, (where they are almost double compared to other similarly developed regions) [1], Blacks in the US and Blacks in the UK. This suggests that the increased risk seen in Blacks in the UK is most likely due to the change in environment in genetically susceptible populations.

The role of genetics in determining variation in risk is reinforced by the observation that both black Africans and Caribbeans display increased prostate cancer incidence, despite different countries of origin; lifestyles and environments.

Consistent with our findings, previous reports have also demonstrated reduced prostate cancer incidence in Asians compared with Whites [28], and within the subset of Asians: Indians, Pakistanis and Bangladeshis [43–47]. The reduced incidence amongst South Asians has been associated with religion, differences in diet, reduced vitamin D levels and early-life sun exposure [48, 49]. Other risk factors associated with prostate cancer are lack of exercise, antioxidants and saturated fat, smoking, alcohol consumption, socioeconomic status and carcinogen exposure (e.g. radiation and arsenic) [50–53] and it is possible that differences in these explain some of the reduced incidence in South Asians. Further, it has been suggested that South Asians meet with more obstacles when accessing health care resources, receiving less diagnostic and screening (PSA) tests [54]. Within South Asian ethnic groups, Bangladeshis displayed a substantially lower incidence of prostate cancer; although the cause of this is likely multifactorial, obesity has been linked to prostate cancer [55], and Bangladeshis show markedly lower obesity prevalence compared to Indians and Pakistanis [26]. Prostate cancer incidence in Chinese was higher than in South Asians and this may in part be contributed to by increased soy, genistein and daidzein intake [56].

The comparison of rates between ethnic groups in England and their countries of origin is problematic for a number of reasons. Firstly, population-based cancer registries simply do not exist in many of these countries, particularly in the areas from where the majority of migrants originate e.g. Punjab & Gujarat in India, Kashmir & Punjab in Pakistan, Sylhet in Bangladesh, Jamaica in the Caribbean and Somalia, Nigeria & Ghana in Africa. and Even where registries exist the quality is very variable and there are differences in cancer registration practices [2]. Rates in these developing countries are also likely to be underestimated due to under-diagnosis and under-ascertainment, and in access to screening and early detection, particularly for prostate cancer [2]. Migrants are also a selective group and may not be representative of the population from which they arose and they may be more or less healthy than the population in their native country [6].

Whilst acknowledging these limitations, the increased incidence of prostate, kidney and testicular cancers in all ethnic groups in England compared to their countries of origin is consistent with changes in environmental risk factors and the differences in screening, diagnosis and registration. For bladder cancer the picture was more mixed with higher incidence seen in the countries of origin of Pakistanis, Black Caribbean and Chinese—the reasons for this are unclear.

The main limitation of our study was that we do not have individual level information on most exposures. Ethnicity information was missing for 25 % of cancer registrations—however, this is lower than previous studies [28, 44] and the sensitivity analysis produced similar results. Recording of ethnicity in HES has improved markedly in the last 20 years, with the percentage of missing ethnicity values falling from 35 % in 1998 to less than 10 % by 2009. The quality of the ethnic coding in HES has also been assessed and no ethnic group is widely misrepresented in HES data for England [13, 14].

Also, the group ‘British White’ inevitably included some ‘Other (non-British) Whites’ as the ethnic category ‘Whites’ included both British Whites and ‘Other Whites’ prior to 2003 although this was less than 5 % of the ‘total White’ category based on the data post-2003 so would not materially affect the results for British Whites. We were therefore also unable to compare British Whites to ‘Other Whites’, and given that the ‘Other White’ population has increased rapidly since 2004 (due to migration from the European Union), future studies should look at incidence in this group as well.

## Conclusions

This is the first and largest study to investigate the differences in incidence of urological cancers by ethnic group in England. The very high incidence of prostate cancer in both black Africans and Caribbeans, despite differing lifestyles and environments and countries of origin is most likely due to the change in environment in genetically susceptible populations. The large differences in incidence we found with other cancers by ethnic group are not readily explained by known risk factors, which suggest that important, potentially modifiable, risk factors are yet to be discovered.

## Additional file

Additional file 1: figure S1. Age-standardised incidence rates and rate ratios (adjusted by age, sex and income) for four urological malignancies by ethnic group, following multiple imputation for missing ethnicity values. (DOC 219 kb)

## Abbreviations

HES: Hospital Episodes Statistics
NCIN: National Cancer Intelligence Network
ICD-10: International Classifications of Diseases, 10th Revision
ICD-O-2: International Classifications of Diseases of Oncology, 2nd Revision
ICD-O-3: International Classifications of Diseases of Oncology, 3rd Revision
ASRs: Age Standardised Rates
IRRs: Incidence Rate Ratios
