# Supplementary figures and images for: Incidence of prostate and urological cancers in England by ethnic group, 2001-2007: a descriptive study
Source: BMC Cancer. 2015 Oct 21;15:753. doi: 10.1186/s12885-015-1771-2 (PMC4618465; doi:10.1186/s12885-015-1771-2)

**
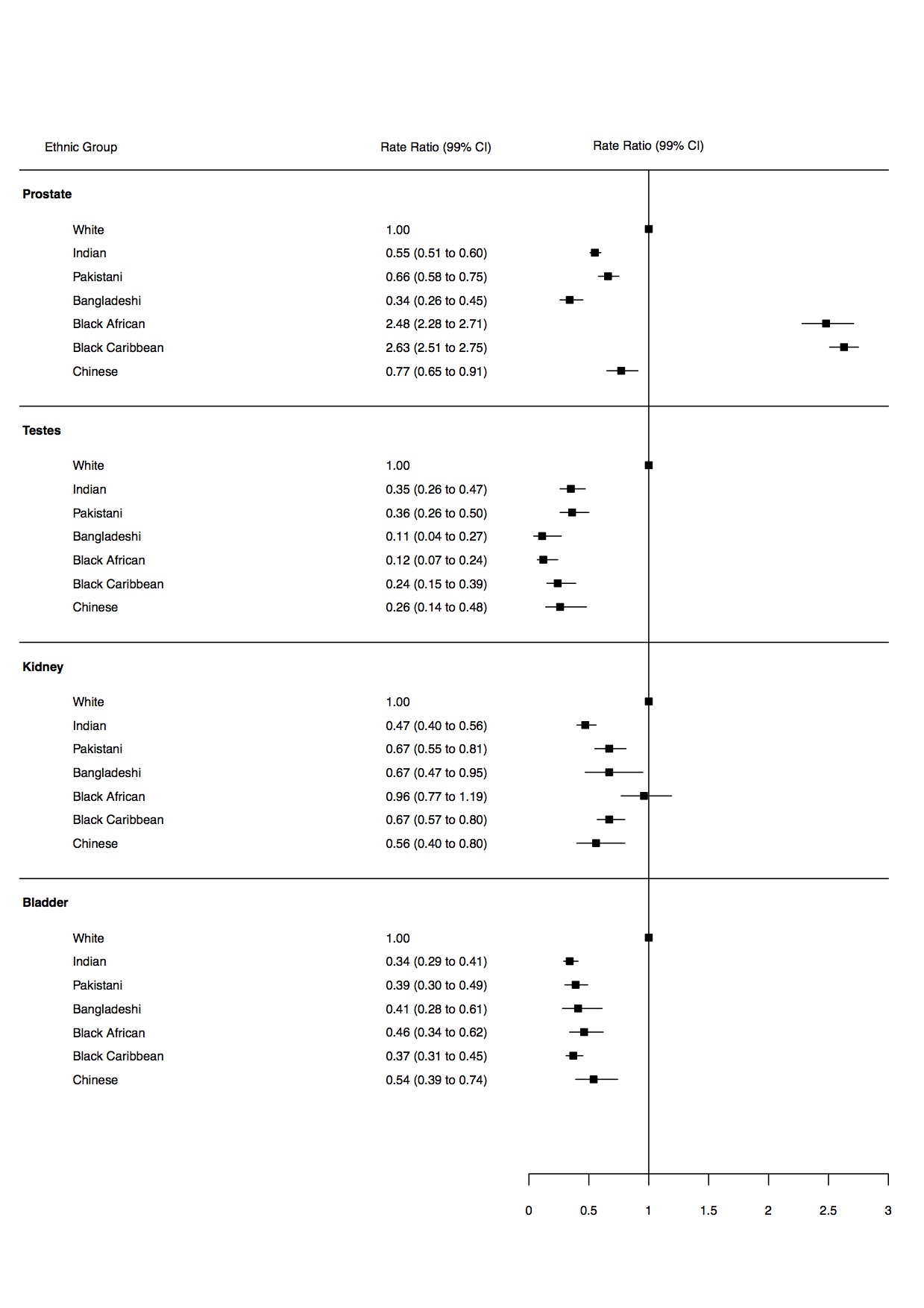
**

Supplement: Additional file 1: figure S1. — Age-standardised incidence rates and rate ratios (adjusted by age, sex and income) for four urological malignancies by ethnic group, following multiple imputation for missing ethnicity values. (DOC 219 kb) [file 12885_2015_1771_MOESM1_ESM.doc]
